# Family and contextual factors associated with licit drug use in adolescence

**DOI:** 10.11606/s1518-8787.2021055003531

**Published:** 2021-11-23

**Authors:** Monalisa Cesarino Gomes, Ana Flávia Granville-Garcia, Erick Tássio Barbosa Neves, Laio da Costa Dutra, Fernanda Morais Ferreira, Saul Martins Paiva

**Affiliations:** I Universidade Federal de Minas Gerais Departamento de Odontopediatria e Ortodontia Belo Horizonte MG Brasil Universidade Federal de Minas Gerais. Departamento de Odontopediatria e Ortodontia. Belo Horizonte, MG, Brasil; II Unifacisa Centro Universitário Departamento de Odontologia Campina Grande PB Brasil Unifacisa Centro Universitário. Departamento de Odontologia. Campina Grande, PB, Brasil; III Universidade Estadual da Paraíba Departamento de Odontologia Campina Grande PB Brasil Universidade Estadual da Paraíba. Departamento de Odontologia. Campina Grande, PB, Brasil

**Keywords:** Adolescent, Drug Users, Underage Drinking, Tobacco Smoking, Risk Factors, Socioeconomic Factors, Family Relations

## Abstract

**OBJECTIVE:**

TO evaluate the family and contextual factors associated with licit drug use among 15 to 19-year-old adolescents in the school context.

**METHODS:**

This is a representative, school-based, cross-sectional study conducted with 746 adolescents from 15 to 19 years old enrolled in public and private schools. Parents/guardians reported on the sociodemographic variables, while adolescents answered questionnaires on drug use, family cohesion and adaptability, oral health literacy and visits to the dentist. Information on school context was obtained at the institution and via municipal publications. Associations between variables were analyzed using unadjusted and adjusted multilevel Poisson regression models.

**RESULTS:**

Prevalence of licit drug use at least once and a pattern indicative of harmful drug use were 39.8% and 15.1%, respectively. After the adjusted analysis of licit drug use at least once, the variables gender (PR = 1.26; 95%CI: 1.01–1.59), family cohesion (PR = 9.81; 95%CI: 1.23–72.54), and average income of the school district (PR = 0.72; 95%CI: 0.57–0.91) remained in the final model. As for drug abuse, only the detached type (PR = 23.01; 95%CI: 2.46–214.87) and separated type (PR = 13.54; 95%CI: 1.40–130.97) of family cohesion remained in the final model.

**CONCLUSION:**

Experience with licit drug use was associated with family and contextual factors among the adolescents, while family cohesion was the main factor related to harmful drug use.

## INTRODUCTION

The transitional stage of development known as adolescence involves a series of transformations that leave teenagers more vulnerable to unhealthy habits. Some behaviors that start at this stage, such as poor diet, drug use, and sedentary lifestyle, are risk factors for the development of chronic noncommunicable diseases such as obesity, cardiovascular disease, and oral problems^1–3^ and determine quality of life in adulthood.

Licit drug use by adolescents is a public health issue that shows high prevalence worldwide^4,5^. In Brazil, the most recent National School Health Survey with basic education students (conducted in 2015) found that 55.5% and 18.4% had experimented with alcohol and cigarettes, respectively. Given such context, the World Health Organization recommends carrying out epidemiological studies to assist in the establishment of public policies, given its status as an important public health strategy^1^.

Another important aspect regarding health-related behavior is family cohesion^6^, defined as the emotional bonds that family members have with each other and the expression of belonging and acceptance within the family^6^. Previous studies already investigated the association between family cohesion and licit drug use, but targeted other age groups^7,9^ or used different questionnaires and drug use categorizations, excluding onset and abuse^7–11^. We posit, therefore, that a higher level of family cohesion is associated with less licit drug use among adolescents.

Besides individual aspects, we must also understand the contextual factors involved in the onset and continued drug use. Contextual assessments allow us to identify risk areas and plan adequate prevention and healthcare strategies at the community level. School is an important context for investigating risk factors, since adolescents spend a large part of their lives in this environment^12^. A study conducted with 15- and 16-year-old adolescents showed that changes in the school environment to promote student participation and improved relationships may be associated with a reduction in drug use^13^.

One can find other multilevel analysis studies on drug use in adolescence^14–18^ in the literature, but these were conducted in different environments^14–18^ and age groups^15,16^, in other countries^15,18^, and involving different categorizations of licit drug use^14–18^. The present study, in turn, proposes a multilevel analysis of both onset drug use and consumption indicative of drug abuse. Such an approach will allow a more detailed assessment of which aspects may be involved in the onset and continuation of this habit. To our knowledge, no previous multilevel analysis study has assessed these two types of drug use in adolescents.

Thus, the present study seeks to evaluate the family and contextual factors associated with licit drug use among 15- to 19-year-old adolescents in the school context.

## METHODS

This study was approved by the Research Ethics Committee of the Paraiba State University (certificate number: 55953516.2.1001.5187) and complied with the Declaration of Helsinki guidelines. Both parents/guardians and adolescents gave written consent.

### Study Design and Sample Size

Conducted in a medium-sized city in northeast Brazil (estimated population: 405,072)^19^ between October 2016 and July 2017, this cross-sectional study involved 15- to 19-year-old adolescents enrolled at public and private schools.

We performed a two-stage probabilistic cluster sampling stratified by district and type of school, resulting in 32 schools (16 public and 16 private) randomly selected based on the distribution of adolescents in the city’s six districts. We then selected the students from each school using a simple randomization procedure, reaching a final number of students proportional to the population in the schools and districts. Sample size calculation followed the standards for analyses comparing the proportions of two independent groups. Using the G* Power software (version 3.1.9.7) and adopting 95% of individuals, of which 35% used licit drugs and had high family cohesion and 49% used licit drugs and had low family cohesion, we obtained a minimum sample of 388 adolescents. After applying a design effect of 1.6 to compensate for the cluster sampling procedure, the sample size reached 621 individuals, to which we added 20% to compensate for possible losses, establishing a final sample of 777 adolescents.

### Eligibility Criteria

Adolescents between the ages of 15 and 19 who could read and write, were enrolled in public or private schools, and had no systemic or cognitive disabilities (based on reports from parents/guardians or teachers) were eligible for this study.

### Pilot Study

We conducted a pilot study with 50 adolescents (25 from a public school and 25 from a private school), selected by convenience and who were not included in the main study. The results showed no need to change the proposed methods.

### Data Collection

#### Individual and family variables

We held meetings with the parents/guardians to explain the objectives of the study and collect the signed informed consent form, after which they answered a questionnaire addressing sociodemographic characteristics (adolescent’s gender, monthly family income [categorized by median], schooling level, marital status, adolescent’s race, and number of residents in the home). The other research instruments were applied to the adolescents at school.

Drug use was assessed using the Alcohol, Smoking and Substance Involvement Screening Test (ASSIST) created by the World Health Organization and validated in Brazil^20,21^. Inquiring about the use of nine classes of psychoactive substances (tobacco, alcohol, marijuana, cocaine, stimulants, sedatives, inhalants, hallucinogens, and opioids), ASSIST consists of eight items that address: frequency of lifetime use and in the last three months, problems related to use, concerns about use by those close to the user, negative impacts on performance of expected tasks, unsuccessful attempts to stop or reduce use, compulsive feelings, and injection drug use. Each response has a corresponding score. The present study evaluated tobacco and alcohol use. For tobacco, a score of 0 to 3 meant occasional use, 4 to 26 indicated abuse, and ≥ 27 suggested dependence. For alcohol, a score of 0 to 10 meant occasional use, 11 to 26 indicated abuse, and ≥ 27 suggested dependence. Both dependent variables (licit drug use sometime in life and harmful substance use) were dichotomized into yes/no answers.

Family adaptability and cohesion was assessed using the Family Adaptability and Cohesion Evaluation Scales (FACES III)^22,23^. Family cohesion was categorized as disengaged (lack of an affective union between family members), separated (moderate affective union between family members), connected (considerable affective union between family members), or enmeshed (maximum affective union between family members). Family adaptability refers to the ability of family members to adjust to changes in family dynamics, being classified as “rigid” (very low adaptability; family follows the rules of a single member and no changes occur in family roles), “structured” (low to moderate adaptability; more role sharing), “flexible” (moderate to high adaptability; more flexibility in terms of control and family rules), or “chaotic” (very high adaptability; undefined roles or leadership)^23^.

#### Contextual variables

The school-based contextual analysis assessed five variables: type of school (public or private), number of adolescents per classroom, school district average monthly income, number of healthcare teams in primary care units in the school district, and sale of alcoholic beverages near the school (within a 100-meter radius). Information on the school district average income was obtained from the Brazilian Institute of Geography and Statistics; the number of healthcare teams in the school district was obtained from the Municipal Secretariat of Health. School data were recorded during the first visit to each institution, based on information provided by the management staff.

### Statistical Analysis

Data organization and statistical analysis were performed using the Statistical Package for the Social Sciences (SPSS for Windows, version 22.0, IBM Inc., Armonk, NY, USA). Sample characterization was accomplished using descriptive statistics. Associations between outcomes and predictors were described by unadjusted and adjusted multilevel Poisson regression models with robust variance correction. Licit drug use sometime in life was assessed first, followed by harmful substance use of the respective drug. The multilevel Poisson regression analysis involved a fixed effects model with random intercepts to assess associations, at each level of analysis, between the outcome and both individual and contextual covariates. This allowed us to estimate the prevalence ratios (PR) between comparison groups and their respective 95% confidence intervals (CI).

First, we used an unconditional (null) model to estimate data variability before including the individual and contextual covariates into each level of analysis. Individual covariates were incorporated into Model 2; both the individual and contextual covariates were incorporated into Model 3. Individual variables that achieved p-value < 0.20 in the bivariate multilevel Poisson regression analysis were incorporated into Model 2, and those who achieved p-value < 0.05 in the adjusted analysis remained in the model. Contextual variables that achieved p-value < 0.20 in the bivariate multilevel Poisson regression analysis were incorporated into Model 3, and those with p-value < 0.05 in the adjusted analysis remained in the final model. Goodness of fit of the models was calculated using deviance values (–2 log likelihood).

## RESULTS

The study included data on 746 pairs of adolescents-parents/guardians, with 31 pairs lost due to incomplete questionnaires, corresponding to a response rate of 96.0%.

Frequency of licit drug use sometime in life among adolescents was 39.8%, with 39.3% having used alcohol and 7.8% tobacco. Frequency of harmful substance use among adolescents was 15.1%, of which 13.9% showed harmful alcohol consumption and 4.7% harmful tobacco use.

Regarding sociodemographic characteristics, most participants were female (59.5%), had a monthly family income of less than R$937 (51.0%), had parents/guardians with high schooling level (51.6%), and attended public school (66.6%).

Table 1 shows the bivariate analysis for licit drug use. Parents’ schooling level, family cohesion, and school district average monthly income were associated with drug use sometime in life (p < 0.05). Family income, parent’s marital status, family cohesion, and type of school were associated with harmful substance use (p < 0.05).

**Table 1:** Unadjusted analysis of associations between licit drug use sometime in life and harmful substance use among adolescents and individual and contextual variables.

Variable			Licit drug use sometime in life			Harmful substance use		
			n%	p	PR (95%CI)	n%	p	PR (95%CI)
Individual level								
	Gender							
		Female	38.5		1.00	14.0		1.00
		Male	41.7	0.194	1.17 (0.92–1.50)	16.9	0.609	1.13 (0.69–1.85)
	Parent/Guardian’s schooling level							
		≤ 8 years of study	41.3	0.015	1.35 (1.06–1.73)	17.1	0.198	1.39 (0.84–2.31)
		> 8 years of study	38.0		1.00	12.7		1.00
	Monthly family income							
		≤ R$937	41.9	0.203	1.20 (0.90–1.59)	17.3	0.015	1.94 (1.13–3.32)
		> R$937	35.2		1.00	11.5		1.00
	Parent/Guardian’s marital status							
		Married	38.2		1.00	12.4		1.00
		Single/Divorced/Widowed	41.5	0.362	1.12 (0.87–1.43)	18.4	0.015	1.81 (1.12–2.93)
	Race							
		White	32.7		1.00	13.3		1.00
		Non-White	42.6	0.163	1.23 (0.91–1.65)	15.9	0.844	1.05 (0.60–1.83)
	Number of residents in home							
		< 6	39.7		1.00	15.0		1.00
		≥ 6	39.2	0.990	1.01 (0.72–1.38)	14.4	0.409	0.76 (0.40–1.44)
	Family cohesion							
		Detached	48.0	0.017	8.85 (1.48–52.67)	20.1	0.002	27.10 (3.35–218.80)
		Separated	35.0	0.060	5.59 (0.93–33.55)	11.7	0.020	12.31 (1.48–102.02)
		Connected	30.6	0.091	4.75 (0.77–29.09)	9.9	0.066	7.67 (0.87–67.25)
		Enmeshed	13.3		1.00	6.7		1.00
	Family adaptability							
		Chaotic/Very flexible	36.8		1.00	20.2		1.00
		Flexible	40.0	0.891	1.02 (0.73–1.42)	14.7	0.162	0.66 (0.37–1.18)
		Structured	42.6	0.935	0.98 (0.69–1.39)	14.4	0.343	0.72 (0.37–1.40)
		Rigid	38.5	0.891	1.02 (0.68–1.54)	10.7	0.609	0.81 (0.36–1.81)
Contextual level: School								
	Type of school							
		Public	42.5	0.093	1.24 (0.96–1.60)	18.7	0.003	2.42 (1.35–4.37)
		Private	34.5		1.00	8.0		1.00
	Number of adolescents in classroom		-	0.190	0.99 (0.97–1.01)	-	0.276	0.98 (0.96–1.01)
	School district average monthly income							
		< monthly minimum wage	33.4	0.017	0.74 (0.58–0.94)	13.7	0.528	0.86 (0.54–1.36)
		≥ monthly minimum wage	44.4		1.00	16.2		1.00
	Number of healthcare teams		-	0.242	0.98 (0.94–1.01)	-	0.927	1.01 (0.94–1.06)
	Sale of alcoholic beverages near school							
		No	40.3	0.234	1.15 (0.91–1.45)	14.9	0.276	1.28 (0.82–1.99)
		Yes	39.1		1.00	15.5		1.00

Note: Total number of participants (n = 746).

In the adjusted multilevel Poisson regression analysis, Model 2 (with only individual variables) showed that being male (PR = 1.29; 95%CI: 1.02–1.62) and having a detached type of family cohesion (PR = 8.37; 95%CI: 1.38–50.80) predicted licit drug use sometime in life. After incorporating the contextual variables, being male (PR = 1.26; 95%CI: 1.01–1.59) and having a detached type of family cohesion (PR = 9.81; 95%CI: 1.23–72.54) remained significant predictors. In the final model (Model 3), school district average monthly income (contextual variable) was associated with licit drug use sometime in life (PR = 0.72; 95%CI: 0.57–0.91), with lower district average income being a protective factor for licit drug use (Table 2).

**Table 2:** Adjusted multilevel analysis of associations between licit drug use sometime in life among adolescents and individual and contextual variables.

Fixed effects			Model 1 (“null”)	Model 2	Model 3
			PR (95%CI)		
Intercept			**0.40 (0.35–0.45)**	**0.04 (0.01–0.26)**	**0.04 (0.01–0.35)**
Individual level					
	Gender				
		Female		**1.00**	**1.00**
		Male		**1.29 (1.02–1.62)**	**1.26 (1.01–1.59)**
	Parent/Guardian’s schooling level				
		≤ 8 years of study		1.21 (0.95–1.54)	1.23 (0.94–1.62)
		> 8 years of study		1.00	1.00
	Race				
		White		1.00	1.00
		Non-White		1.23 (0.93–1.62)	1.25 (0.95–1.66)
	Family cohesion				
		Detached		**8.37 (1.38–50.80)**	**9.81 (1.23–72.54)**
		Separated		**5.63 (0.92–34.34)**	**6.73 (0.90–50.24)**
		Connected		**5.16 (0.83–32.14)**	**5.80 (0.76–44.20)**
		Enmeshed		**1.00**	**1.00**
Contextual level: school					
	Type of school				
		Public			0.92 (0.67–1.27)
		Private			1.00
	School district average monthly income				
		< monthly minimum wage			**0.72 (0.57–0.91)**
		≥ monthly minimum wage			**1.00**
	Number of adolescents in classroom				0.99 (0.98–1.01)
		Random effects			
		Deviance (-2 log likelihood)	57,679,175	52,727,713	50,603,679

Model 1 (“null”): unconditional model; Model 2: individual covariates; Model 3: individual and contextual covariates.

Note: Total number of participants (n = 746).

In the adjusted multilevel Poisson regression analysis for harmful substance use, Model 2 (with only individual variables) found associations only with family cohesion, with the detached type (PR = 23.73; 95%CI: 2.59–216.86) and separated type (PR = 13.18; 95%CI: 1.40–123.76) being risk factors for this consumption pattern. After incorporating a contextual variable (type of school) (Model 3), having a detached type (PR = 23.01; 95%CI: 2.46–214.87) and separated type (PR = 13.54; 95%CI: 1.40–130.97) of family cohesion remained associated, while type of school was not significant. After the final adjustment, only family cohesion remained significantly associated with harmful substance use among adolescents (Table 3).

**Table 3:** Adjusted multilevel analysis of associations between harmful substance use among adolescents and individual and contextual variables.

Fixed effects			Model 1 (“null”)	Model 2	Model 3
			PR (95%CI)		
Intercept			0.16 (0.12–0.21)	0.01 (0.01–0.09)	0.01 (0.01–0.07)
Individual level					
	Monthly family income				
		≤ R$937		1.48 (0.82–2.69)	1.15 (0.66–1.99)
		> R$937		1.00	1.00
	Parent/Guardian’s schooling level				
		≤ 8 years of study		0.98 (0.56–1.73)	0.84 (0.47–1.50)
		> 8 years of study		1.00	1.00
	Parent/Guardian’s marital status				
		Married		1.00	1.00
		Single/Divorced/Widowed		1.49 (0.86–2.57)	1.39 (0.82–2.35)
	Family cohesion				
		Detached		**23.73 (2.59–216.86)**	**23.01 (2.46–214.87)**
		Separated		**13.18 (1.40–123.76)**	**13.54 (1.40–130.97)**
		Connected		**7.13 (0.67–75.61)**	**7.65 (0.70–82.95)**
		Enmeshed		**1.00**	**1.00**
	Family adaptability				
		Chaotic/Very flexible		1.00	1.00
		Flexible		0.66 (0.35–1.25)	0.67 (0.36–1.26)
		Structured		0.66 (0.33–1.31)	0.64 (0.32–1.29)
		Rigid		0.77 (0.37–1.58)	0.76 (0.37–1.54)
Contextual level: School					
	Type of school				
		Public			2.10 (0.95–4.64)
		Private			1.00
		Random effects			
		Deviance (-2 log likelihood)	34,557,746	23,598,829	23,345,729

Model 1 (“null”): unconditional model; Model 2: individual covariates; Model 3: individual and contextual covariates.

Note: Total number of participants (n = 746).

## DISCUSSION

Our study assessed the individual and contextual predictors of licit drug use among adolescents. Being male, having a family with low family cohesion, and studying in a school district with high average income were significantly associated with licit drug use, while only family cohesion was associated with harmful substance use. These results confirm our hypothesis that higher levels of family cohesion would predict lower licit drug use among adolescents. Based on such findings, we can affirm that licit drug use among adolescents is a complex issue that involves individual (gender), family (family cohesion), and social (school) factors^15^.

We found a greater frequency of licit drug use sometime in life among male adolescents than females. Such high prevalence may be explained by the cultural norms attributed to gender by society^25^, but previous studies with adolescents show no consensus regarding the association between gender and drug use^12,24,25^. Harmful drug use, however, showed no association with gender, only with family cohesion. Thus, although gender is associated with onset use, family aspects seem to have more influence on continued use, since good family relations contribute to establish healthy habits. Adolescence is a period of biopsychosocial changes and adolescents are prone to new experiences, regardless of gender^1^.

Adolescents with low family cohesion (detached and separated types) had a higher prevalence of both licit drug use sometime in life and harmful drug use. A study conducted in Puerto Rico involving 18- to 64-year-old individuals found that greater family cohesion was a protective factor for drug use^7^. Good family cohesion can facilitate communication between family members and improve family functioning. One study suggested that family cohesion could serve as a protective factor for alcohol use disorders^9^. An unstable family environment lacks resources for social support, which hinders the ability to establish healthy relationships, affecting both physical and mental health^26^.

Regarding social factors, the literature reports conflicting results on the association between socioeconomic status and licit drug use among adolescents^18,24,25^. In the present study, while family income showed no association with drug use, the school district’s average monthly income was associated with licit drug use, as the frequency of drug use sometime in life was greater in districts with high average income. As a contextual socioeconomic aspect, most families in better socioeconomic status neighborhoods may be wealthier. One study showed that teenagers from wealthier families have greater access to disposable income and feel safer experimenting with alcohol or tobacco^27^. Increased buying power combined with the popularization of different alcohol and tobacco options may thus favor drug use as a form of social belonging and interaction. The school district’s average income was not associated with harmful drug use, showing the importance of the family environment for continued use. A systematic review evaluating the influence of residential neighborhood socioeconomic status on drug use suggested that contextual socioeconomic status is more related to drug use among adults, while drug use among adolescents may not result from stress induced by exposure to economically deprived environments^28^. Thus, family relations involving parent-child communication regarding drug use can play a central role in preventing the use of these substances in adolescence.

The cross-sectional design of the study limited establishing cause-and-effect relations. Drug use analysis was based on self-reported information, which is subject to information bias in the form of underreporting. However, the study used a calculated sample size and validated self-administered instruments in a reserved room to ensure anonymity, thus allowing a proper analysis according to the STROBE initiative guidelines.

Our findings highlight the importance of family cohesion and school context in alcohol and tobacco use among adolescents. Licit drug use was associated with family cohesion and school district income, while harmful substance use was influenced primarily by family environment. Thus, the family environment is essential for establishing healthy habits. Public policies should aim at strengthening family-based information and promoting healthy behaviors. Health education actions in schools are also important and should include the participation of families in an attempt to prevent licit drug use.

## References

[1] Viner RM, Ross D, Hardy R, Kuh D, Power C, Johnson A, et al. Life course epidemiology: recognising the importance of adolescence. J Epidemiol Community Health. 2015;69(8):719-20. https://doi.org/10.1136/jech-2014-205300 10.1136/jech-2014-205300 PMC4515995 25646208

[2] Malta DC, Moura L, Prado RR, Escalante JC, Schmidt MI, Duncan BB. Mortalidade por doenças crônicas não transmissíveis no Brasil e suas regiões, 2000 a 2011. Epidemiol Serv Saude. 2014;23(4):599-608. https://doi.org/10.5123/S1679-49742014000400002 10.5123/S1679-49742014000400002

[3] Pinto-Filho JM, Ribeiro LSF, Sartori L, Santos JN, Ramalho LMP, Cury PR. Association between alcohol dependence and both periodontal disease and tooth loss: a cross-sectional study. Environ Sci Pollut Res Int. 2018;25(29):29089-95. https://doi.org/10.1007/s11356-018-2807-3 10.1007/s11356-018-2807-3 30112640

[4] Instituto Brasileiro de Geografia e Estatística. PeNSE - Pesquisa Nacional de Saúde do Escolar. Rio de Janeiro: IBGE; 2016.

[5] ESPAD Group. ESPAD Report 2015: results from the European School Survey Project on Alcohol and Other Drugs. Lisbon (PT): The European Monitoring Centre for Drugs and Dug Addiction-EMCDDA: 2016.

[6] Ferreira LL, Brandão GAM, Garcia G, Batista MJ, Costa LST, Ambrosano GM, et al. [Family cohesion associated with oral health, socioeconomic factors and health behavior]. Cienc Saude Coletiva. 2013;18(8):2461-73. Portuguese. https://doi.org/10.1590/s1413-81232013000800031 10.1590/s1413-81232013000800031 23896929

[7] Caetano R, Vaeth PAC, Canino G. Family cohesion and pride: drinking and alcohol use disorders in Puerto Rico. Am J Drug Alcohol Abuse. 2017;43(1):87-94. https://doi.org/10.1080/00952990.2016.1225073 10.1080/00952990.2016.1225073 PMC5597303 27808561

[8] Laghi F, Bianchi D, Pompili S, Lonigro A, Baiocco, R. Binge eating and binge drinking behaviors: the role of family functioning. Psychol Health Med. 2021;26(4):408-20. https://doi.org/10.1080/13548506.2020.1742926 10.1080/13548506.2020.1742926 32228049

[9] Cano MA, Sánchez M, Rojas P, Ramírez-Ortiz D, Polo KL, Romano E, et al. Alcohol use severity among adult Hispanic immigrants: examining the roles of family cohesion, social support and gender. Subst Use Misuse. 2018;53(4):668-76. https://doi.org/10.1080/10826084.2017.1356333 10.1080/10826084.2017.1356333 PMC5820212 28910173

[10] Pourramazani N, Sharifi H, Iranpour A. Social capital and its relationship with drug use among Southeast Iranian adolescents. Addict Health. 2019;11(1):58-65. https://doi.org/10.22122/ahj.v11i1.230 10.22122/ahj.v11i1.230 PMC6612233 31308911

[11] Shih RA, Parast L, Pedersen ER, Troxel WM, Tucker JS, Miles JNV, et al. Individual, peer and family factor modification of neighborhood-level effects on adolescent alcohol, cigarette, e-cigarette and marijuana use. Drug Alcohol Depend. 2017;180(1):76-85. https://doi.org/10.1016/j.drugalcdep.2017.07.014 10.1016/j.drugalcdep.2017.07.014 PMC5693315 28886395

[12] Freitas EAO, Martins MSAS, Espinosa MM. Alcohol and tobacco experimentation among adolescents of the Midwest Region/Brazil. Cienc Saude Coletiva. 2019;24(4):1347-57. https://doi.org/10.1590/1413-81232018244.15582017 10.1590/1413-81232018244.15582017 31066837

[13] Busch V, De Leeuw RJJ, Schrijvers AJP. Results of a multibehavioral health-promoting school pilot intervention in a Dutch secondary school. J Adolesc Health. 2013;52(4):400-6. https://doi.org/10.1016/j.jadohealth.2012.07.008 10.1016/j.jadohealth.2012.07.008 23299009

[14] Andersen CS, Horta RL, Pattussi MP. Access to information in school and the use of psychoactive substances in Brazilian students: a multilevel study. Addict Behav Rep. 2018;8(1):66-70. https://doi.org/10.1016/j.abrep.2018.07.004 10.1016/j.abrep.2018.07.004 PMC6071580 30094325

[15] Lung Y, Chang SS, Hsu CY, Wu SC, Chen CY, Chen WJ. Residential socioeconomic environments and areca nut use in Taiwan: a comparison with alcohol and tobacco use in multilevel analysis. Subst Use Misuse. 2020;55(12):2025-34. https://doi.org/10.1080/10826084.2020.1788089 10.1080/10826084.2020.1788089 32654584

[16] Leal-López E, Moreno-Maldonado C, Inchley J, Deforche B, Van Havere T, Van Damme J, et al. Association of alcohol control policies with adolescent alcohol consumption and with social inequality in adolescent alcohol consumption: a multilevel study in 33 countries and regions. Int J Drug Policy. 2020;84:102854. https://doi.org/10.1016/j.drugpo.2020.102854 10.1016/j.drugpo.2020.102854 PMC7762782 32717703

[17] Paz FM, Teixeira VA, Pinto RO, Andersen CS, Fontoura LP, Castro LC, et al. School health promotion and use of drugs among students in Southern Brazil. Rev Saude Publica. 2018;52:58. https://doi.org/10.11606/s1518-8787.2018052000311 10.11606/s1518-8787.2018052000311 PMC5958966 29791679

[18] Gaete J, Araya R. Individual and contextual factors associated with tobacco, alcohol and cannabis use among Chilean adolescents: a multilevel study. J Adolesc. 2017;56:166-78. https://doi.org/10.1016/j.adolescence.2017.02.011 10.1016/j.adolescence.2017.02.011 28259098

[19] Instituto Brasileiro de Geografia e Estatística. Censo 2010: resultados. Rio de Janeiro: IBGE: c2021 [cited 2021 Mar 1]. Available from: https://censo2010.ibge.gov.br/resultados.html

[20] Henrique IFS, Micheli D, Lacerda RB, Lacerda LA, Formigoni MLOS. [Validation of the Brazilian version of Alcohol, Smoking and Substance Involvement Screening Test (ASSIST)]. Rev Assoc Med Bras (1992). 2004;50(2):199-206. Portuguese. https://doi.org/10.1590/s0104-42302004000200039 10.1590/s0104-42302004000200039 15286871

[21] WHO ASSIST Working Group. The Alcohol, Smoking and Substance Involvement Screening Test (ASSIST): development, reliability and feasibility. Addiction. 2002;97(9):1183-94. https://doi.org/10.1046/j.1360-0443.2002.00185.x 10.1046/j.1360-0443.2002.00185.x 12199834

[22] Olson DH, Russel CS, Sprenkle DH, editors. Circumflex model: systemic assessment and treatment of families. New York: The Harworthpress; 1989.

[23] Falceto OG, Busnello ED, Bozzetti MC. [Validation of diagnostic scales of family functioning for use in primary health care services]. Rev Panam Salud Publica. 2000;7(4):255-63. Portuguese. https://doi.org/10.1590/s1020-49892000000400007 10.1590/s1020-49892000000400007 10846929

[24] Díaz Geada A, Busto Miramontes A, Caamaño Isorna FC. Alcohol, tobacco and cannabis consumption in adolescents from a multicultural population (Burela, Lugo). Adicciones. 2018;30(4):264-70. https://doi.org/10.20882/adicciones.915 10.20882/adicciones.915 29353293

[25] Mutumba M, Schulenberg JE. Tobacco and alcohol use among youth in low and middle income countries: a multi-country analysis on the influence of structural and micro-level factors. Subst Use Misuse. 2019;54(3):396-411. https://doi.org/10.1080/10826084.2018.1497063 10.1080/10826084.2018.1497063 PMC6438732 30654696

[26] Hakulinen C, Pulkki-Raback L, Jokela M, Ferri JE, Aalto AM, Virtanen M, et al. Structural and functional aspects of social support as predictors of mental and physical health trajectories: Whitehall II cohort study. J Epidemiol Community Health. 2016;70(7):710-5. https://doi.org/10.1136/jech-2015-206165 10.1136/jech-2015-206165 26767407

[27] Martin BA, McCoy TP, Champion H, Parries MT, DuRant RH, Mitra A, et al. The role of monthly spending money in college student drinking behaviors and their consequences. J Am Coll Health. 2009;57(6):587-96. https://doi.org/10.3200/JACH.57.6.587-596 10.3200/JACH.57.6.587-596 19433396

[28] Karriker-Jaffe KJ. Areas of disadvantage: a systematic review of effects of area-level socioeconomic status on substance use outcomes. Drug Alcohol Rev. 2011;30(1):84-95. https://doi.org/10.1111/j.1465-3362.2010.00191.x 10.1111/j.1465-3362.2010.00191.x PMC3057656 21219502

